# Molecular Analysis of a Leprosy Immunotherapeutic Bacillus Provides Insights into *Mycobacterium* Evolution

**DOI:** 10.1371/journal.pone.0000968

**Published:** 2007-10-03

**Authors:** Niyaz Ahmed, Vikram Saini, Saurabh Raghuvanshi, Jitendra P. Khurana, Akhilesh K. Tyagi, Anil K. Tyagi, Seyed E. Hasnain

**Affiliations:** 1 Pathogen Evolution Laboratory, Centre for DNA Fingerprinting and Diagnostics (CDFD), Hyderabad, India; 2 Interdisciplinary Centre for Plant Genomics, Department of Plant Molecular Biology, University of Delhi, New Delhi, India; 3 Department of Biochemistry, University of Delhi, New Delhi, India; 4 University of Hyderabad, Hyderabad, India; 5 Institute of Life Sciences, University of Hyderabad, Hyderabad, India; 6 Jawaharlal Nehru Centre for Advanced Scientific Research, Jakkur, Bangalore, India; Aga Khan University, Pakistan

## Abstract

**Background:**

Evolutionary dynamics plays a central role in facilitating the mechanisms of species divergence among pathogenic and saprophytic mycobacteria. The ability of mycobacteria to colonize hosts, to proliferate and to cause diseases has evolved due to its predisposition to various evolutionary forces acting over a period of time. *Mycobacterium indicus pranii* (*MIP*), a taxonomically unknown ‘generalist’ mycobacterium, acts as an immunotherapeutic against leprosy and is approved for use as a vaccine against it. The large-scale field trials of this *MIP* based leprosy vaccine coupled with its demonstrated immunomodulatory and adjuvant property has led to human clinical evaluations of *MIP* in interventions against HIV-AIDS, psoriasis and bladder cancer. *MIP*, commercially available as ‘Immuvac’, is currently the focus of advanced phase III clinical trials for its antituberculosis efficacy. Thus a comprehensive analysis of *MIP* vis-à-vis evolutionary path, underpinning its immanent immunomodulating properties is of the highest desiderata.

**Principal Findings:**

Genome wide comparisons together with molecular phylogenetic analyses by fluorescent amplified fragment length polymorphism (FAFLP), enterobacterial repetitive intergenic consensus (ERIC) based genotyping and candidate orthologues sequencing revealed that *MIP* has been the predecessor of highly pathogenic *Mycobacterium avium intracellulare* complex (*MAIC*) that did not resort to parasitic adaptation by reductional gene evolution and therefore, preferred a free living life-style. Further analysis suggested a shared aquatic phase of *MAIC* bacilli with the early pathogenic forms of *Mycobacterium*, well before the latter diverged as ‘specialists’.

**Conclusions/Significance:**

This evolutionary paradigm possibly affirms to marshal our understanding about the acquisition and optimization of virulence in mycobacteria and determinants of boundaries therein.

## Introduction

Genus *Mycobacterium* represents some of man's most potent microbiological adversaries like *M. tuberculosis* (*MTB*), the tuberculosis (TB) causing bacterium that is responsible for the loss of more than 50,000 human lives every week, globally. Also, leprosy caused by *M. leprae*, is still a major public health problem [1] whereas *M. avium* and other ‘opportunist’ mycobacteria are the major cause of disease and death in immune compromised hosts, including HIV patients. Moreover, the advent of XDR [2] and MDR [3] strains of *MTB* coupled to prevalence of HIV co-infection and the emergence of TB-IRIS (Immune Responsive Inflammatory Syndrome) [4], has exacerbated the situation. Despite better insights into the molecular basis of disease pathogenesis, substantial gaps persist in our understanding of the evolution of the soil-derived mycobacterial progenitors into ‘seasoned’ pathogens, and their effective prophylaxis.

*Mycobacterium indicus pranii* (*MIP)*, formerly *Mycobacterium w* [5], is an atypical saprophytic bacterium that was listed in Runyon Group IV, along with *M. fortuitum*, *M. smegmatis*, *M. chelonae* and *M. vaccae*, based on its growth and metabolic properties [6]. *MIP*, in an extended phase III clinical trial in India, was used as an adjunct to the standard multidrug therapy with multibacillary leprosy patients and showed a significantly enhanced bacillary clearance, thus, shortening the full recovery time [6], [7]. *MIP*, commercially available as “Immuvac” vaccine, also exhibited immunoprophylactic benefits in household contacts of leprosy patients in the largest ever clinical trial in India [8]. It not only shares antigens with *M. leprae* and *M. tuberculosis* but also provides protection to both BCG responder and non-responder genetic strains of mice against *M. tuberculosis* H37Rv infection [9]. Based on a strong indication of its immuno-therapeutic role in category II tuberculosis patients [10], large-scale phase III trials are currently in progress to evaluate its anti-tuberculosis efficacy. *MIP* is also under clinical trials as an immuno-modulator and adjuvant, based on encouraging findings about its role in HIV [11], bladder cancer [12] and psoriasis [13].

To optimally harness the therapeutic potential of *MIP*, it is imperative to understand how *MIP* shaped its exceptional immunomodulating properties akin to a philanthropic vaccine strain without embracing the dreadful pathogenic attributes of *MTB*. Conventionally, evolutionary and comparative genomic studies have been Rosetta stone to gain insights into mycobacterial divergence and acquisition of virulence therein. The epistemological studies involving extensive genomic characterization of *MIP* using several molecular tools and markers along with the comparative genomic studies with its whole genome data reveal that *MIP* has been the predecessor of *MAIC* bacilli and shared a common aquatic phase with early pathogenic forms of mycobacteria thus, presenting a holistic picture of *Mycobacterium* evolution.

## Results

### MIP belongs to MAIC

**Figure 1:**
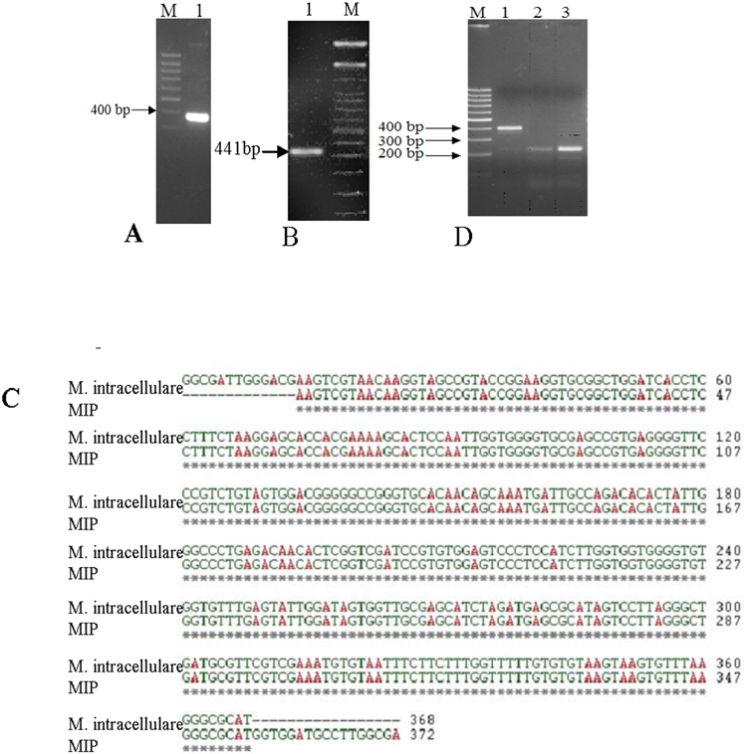
Confirmation of the genetic signatures of *MIP* (A) PCR with ITS region sequences showing a ∼350 bp amplicon, M-100 bp marker (B) *MIP* strain confirmation PCR with *hsp65* gene region, M-100 bp marker. This 441 bp amplicon was having all the 4 unique substitutions known for *MIP* (C) Sequence alignment of ITS sequence of *MIP* with *MAIC* organism (D) IS900 PCR to rule out *MAPC* lineage-M: marker, Lane 1: *M. paratuberculosis* control PCR, lanes 2 and 3: *MIP.*

*MIP* genomic DNA was subjected to different diagnostic PCRs targeted at signature sequences such as internal transcribed spacer (ITS) region between *rrn16* and *rrn23* genes and 65 kDa heat shock protein (*hsp*65) gene (Figure 1). The amplicons corresponding to these loci were sequenced and aligned to evaluate sequence similarity of *MIP* with other mycobacterial bacilli. The ITS region matched perfectly well with the corresponding region of *M*. *intracellulare* (Figure 1C) pointing to the possible genetic affinity of *MIP* to *MAIC* complex. In *hsp*65, all the four nucleotide substitutions, reported as *MIP* signatures, were identified at the right locations, thereby confirming the identity of *MIP* used in this study [14]. The IS900 specific PCR, considered as a signature for *MAPC* (*M. avium* subsp. *paratuberculosis* complex) bacilli, however, did not yield PCR product specific to *MAPC* (Figure 1), thereby excluding an evolutionary link between *MIP* and *MAPC*. The three principle genetic groups (PGG) based on the single nucleotide polymorphisms of *kat*G 463CTG (Leu) and *gyr*A 95ACC (Thr) (15) showed that *MIP* and *MAIC* bacilli belong to group 1 (primitive) that includes pathogenic branch members like *M. marinum*, *M. ulcerans* and *MTB* W-Beijing strain.

### Phylogenetic placement

**Figure 2:**
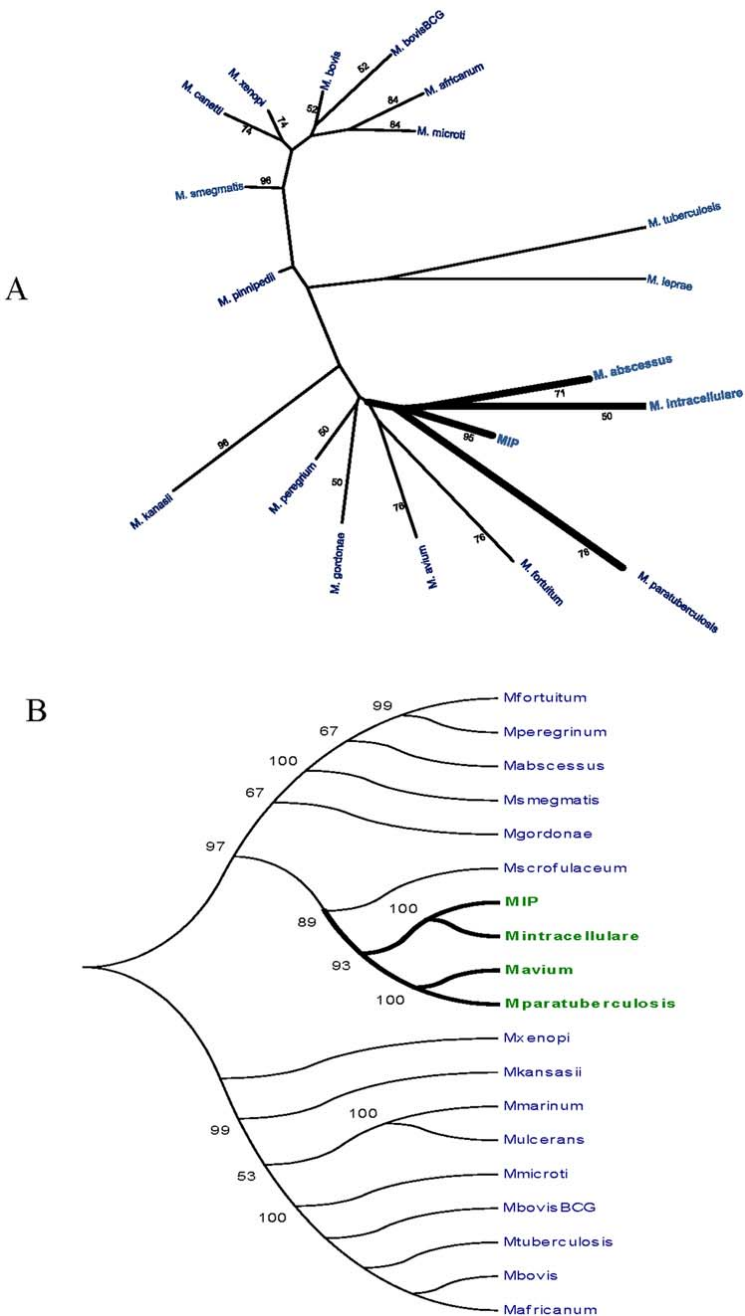
Phylogenetic trees based on FAFLP and multigene sequence analyses. A. Polymorphic fragments were subjected to allele calling in Genotyper (Applied Biosystems, USA) and allele scoring was recorded in a binary format. This binary data were used to construct a phylogenetic tree (please see methods). B. Phylogenetic tree constructed based on consensus multigene alignment which involved concatenation of individual gene sequences corresponding to *rpo*B, *rec*A, *sod*A, *rrn*16 and *hsp*65. Bootstrap values conveying significance of the internal branch topology are clearly marked near each branch. Values above 50 were deemed to be significant to convey correct topology of the internal branches.

**Figure 3:**
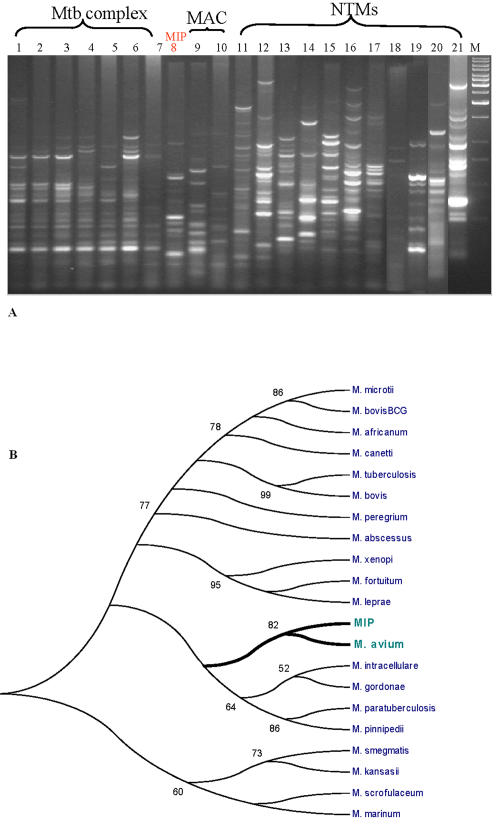
ERIC analysis of *MIP,* other tubercle bacilli and NTMs. A. ERIC based fingerprinting. B. Phylogenetic analysis. Polymorphic ERIC fragments were subjected to allele calling in Quantity 1 software (Biorad, USA) and scoring was recorded in a binary format. These binary data were used to construct a phylogenetic tree developed using bootstrapping methods in MEGA software. Bootstrap values for the internal branch topology are clearly marked near each branch. Values above 50 were assumed as significant to convey acceptable topology of the internal branches.

FAFLP [16] and ERIC [17] are the whole genome based cardinal genotyping approaches that complement the piecemeal studies with candidate genes that may be influenced by horizontal gene transfer events in closely related species. FAFLP analysis (Figure 2A) as well as ERIC based molecular typing revealed considerable genetic similarity of *MIP* with *MAIC* (Figure 3). *MIP*, along with the members of *MAIC*, formed a separate group. This method did not reveal any significant genetic similarity between the bacilli belonging to *M. tuberculosis* complex (*MTBC*) and *MIP.* All the pathogenic species, including *M. leprae,* formed a separate cluster. *MIP* was found to be genetically linked to *M. intracellulare* and *M. abcessus*. All the candidate gene sequences upon alignment with known databank sequences of *MTBC*, *MAPC*, *MAIC* and other non-tuberculous mycobacteria (NTMs), revealed substantial sequence similarities between *MIP* and *MAC* as well as *MAIC* bacilli. Construction of a phylogenetic tree based on concatenated multigene “super locus” comprising of sequences derived from *rrn*16, *sod*A, *hsp*65, *rpo*B and *rec*A eventually placed *MIP* into *MAIC* cluster [18]. *MIP* DNA sequences corresponding to candidate orthologues have been deposited to Genbank (DQ437715-437722).

The phylogeny with *gyr*B [19] and 32 kDa protein genes [20], which are crucial to infer evolutionary relationship in closely related species also confirmed the above findings (Figure S1). The 32 kDa protein coding gene, despite having an identical sequence in strains belonging to *M. tuberculosis* complex (*M*. *tuberculosis*, *M. bovis, M. bovis* BCG and *M. microti*), considerably differs at the nucleotide level in case of *M. avium* and *M. intracellulare* [20]. Likewise, *gyrB* gene is a house-keeping gene, found in almost all bacteria and does not appear to be frequently horizontally transmitted [21]. The alignment of *gyr*B of *MAIC* bacilli and *MIP* showed just one substitution in *MIP* sequence with respect to *M. intracellulare* as compared to 119 of *MAP* (Figure S2). This almost 100% conservation of *gyr*B between *MIP* and *M. intracellulare* is surprising since *gyr*B evolves rapidly due to its faster evolution rate. In case of 32 kDa protein coding gene, similar analysis of *MIP* with that of *M. avium* complex revealed 98% similarity with *M*. *intracellulare* as compared to 93% of *MAP* (Figure S3), thus firmly establishing phylogenetic proximity of *MIP* to *Mycobacterium intracellulare*.

### Evolution of mycobacterial ‘generalists’ versus ‘specialists’

The comparative genome analyses have been employed to dissect the intricate mechanisms of ancient and contemporary dissemination and evolution of mycobacteria [22], [23]. Considering their broadly clonal population structure, it has been hypothesized that a single strain may not have a sufficiently divergent genetic material to qualify for a species status and, therefore, speciation in *Mycobacterium* most likely has arisen due to specific deletion events [22]. Lately, several genomic regions of deletions (RDs), considered as an important evolutionary paradigm in mycobacteria, have been shown to be associated with the attenuation of virulence [23], [24]. The analyses revealed that *MIP* has shown a congruent pattern of RD's with respect to the members of *MAIC* and a similar pattern with that of early pathogenic members of *MTBC* like *M. marinum* and *M. ulcerans*. The RD1 locus, encoding ESAT6 and CFP10 proteins putatively associated with virulence, was absent in *MIP* and *MAIC* bacilli, but present in *M. marinum*. While RD4 is absent in *M. ulcerans*, it is only partially deleted in *M. marinum*. In *M. ulcerans*, ESAT6 and CFP10, known to enhance DNA transfer, are deleted [25]. Acquisition of plasmids in the early group of bacilli, with some deficient in RD1, appears to be a faster mode to gain novel functions for diversification as exemplified by similar synonymous substitution frequencies for plasmid and chromosomal encoded genes in *M. ulcerans* [26]. Likewise, RD3 and RD11 are absent in *MIP* and *MAIC* as well as in *M. marinum*, *M. ulcerans* and *M. canetti,* but RD2 is present in *M. canetti* (Table 1). Similarly, *MIP* and *MAIC* bacilli can be typified by the absence of RD6 region. It is noteworthy that RD6 constitutes IS1532 transposase integrations while RD3 and RD11 represents phiRv phage based integrations into the genomes that may be helpful to generate antigenic diversities. The presence of these regions in the subsequent *MTBC* members, however, suggests that these were deleted only in some lineages of tubercle bacilli. Alternatively, these regions might have been regained via phiRv phage based transductions in recently evolved *MTB*C members.

**Table 1:** Distribution of Regions of deletion (RDs) across the Mycobacterial ‘specialists’ and ‘generalists’

Region	*M. marinum*	*M. ulcerans*	*M. canetti*	*M. africanum*	*M. microti*	*M. bovis*	*M. bovis BCG*	*M.tb H37Rv*	*M. smegmatis*	*MIP*	*MAP*	*MAA*
RD1	Present	Partially present	Present	Present	Present	Present	Absent	Present	Present	Absent	Absent	Absent
RD2	Partially present	Partially present	Present	Present	Present	Present	Absent	Present	Absent	Partially present	Partially present	Partially present
RD3	Absent	Absent	Absent	Absent	Present	Absent	Present	Present	Absent	Absent	Absent	Absent
RD4	Partially present	Absent	Present	Present	Present	Absent	Absent	Present	Absent	Partially present	Partially present	Partially present
RD5	Present	Present	Present	Present	Absent	Absent	Absent	Present	Absent	Partially present	Partially present	Partially present
RD6	Absent	Absent	Present	Present	Absent	Absent	Absent	Present	Absent	Absent	Absent	Absent
RD7	Present	Present	Present	Present	Absent	Absent	Absent	Present	Partially present	Partially present	Partially present	Partially present
RD8	Present	Present	Present	Present	Absent	Absent	Absent	Present	Partially present	Present	Present	Present
RD9	Present	Present	Present	Absent	Absent	Absent	Absent	Present	present	Present	Present	Present
RD10	Present	Present	Present	Present	Absent	Absent	Absent	Present	Present	Present	Present	Present
RD11	Absent	Absent	Absent	Present	Present	Absent	Present	Present	Absent	Absent	Absent	Absent
RD12	Partially present	Partially present	Present	Present	Present	Absent	Present	Present	Partially present	Partially present	Partially present	Partially present
RD13	Present	Present	Present	Present	Present	Absent	Present	Present	Present	Present	Present	Present
RD14	Partially present	Partially present	Present	Present	Present	Present	Present	Present	Absent	Absent	Absent	Absent
RD15	Partially present	Partially present	Not studied	Not studied	Not studied	Absent	Present	Present	Absent	Partially present	Partially present	Partially present
RD16	Present	Present	Not studied	Not studied	Not studied	Absent	Present	Present	Present	Present	Present	Present

**Figure 4:**
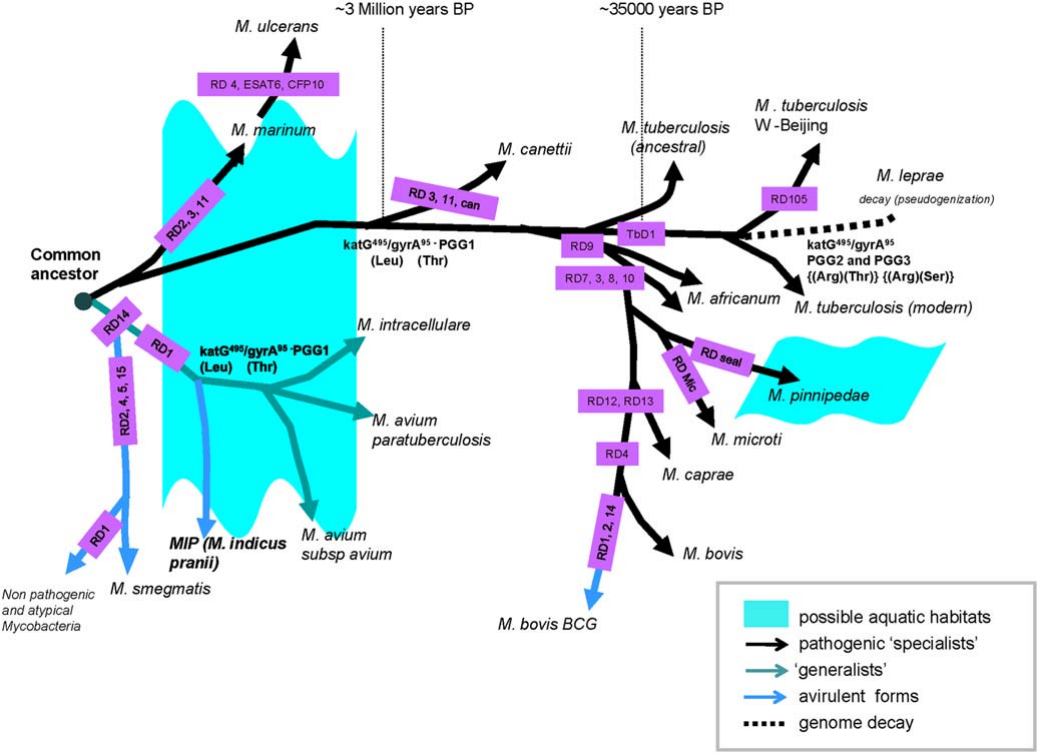
Landscape of genome evolution across the ‘generalist’ and ‘specialist’ mycobacterial lineages. Various RDs refer to genomic deletion points as discussed elsewhere [24]. Divergence of *M. tuberculosis* W-Beijing was deduced based on the RD105 [41]. The inferred time points of some important lineage divergences mentioned as years-before-present (years BP) on the basis of the conventions established earlier [15], [24], [42]. For further details please refer to manuscript text.

Furthermore, the evaluation of *MIP*, based on whole genome data at 10X coverage (Saini *et al*, unpublished), revealed that its genome content is ∼20% in excess to that of *M. avium*. The phylogenetic data in light of genome size evaluation placed *MIP* way ahead of *MAIC* bacilli on an evolutionary time scale (Figure 4). Also, *M. marinum*, in pathogenic branch, and *M. smegmatis* together with *MIP*, within the ‘generalist’ branch, appear as the immediate descendants of the ancient mycobacterial progenitor (Figure 4)**.**

## Discussion

The saprophytic mycobacteria are believed to play a protective role in chronic infections like asthma and TB [27]. However, clinical trials with saprophytic *M. vaccae* didn't benefit TB patients and led to local adverse reactions in recipients [28]. Contrarily, *MIP*, besides giving protection against TB and AIDS infections, elicits immune responses even in the heat inactivated form [29], [30]. Considering variable BCG efficacy and increasing HIV/TB pandemic, alternate strategies involving *MIP* are pertinent to confront this deadly duo that has caused nearly 150 million deaths since World War I-more total deaths than in all wars in the last 2000 years [31].

The phylogenetic link of saprophytic *MIP* to the non-tubercle *MAIC* bacilli came as a surprise especially as all the members of this ilk are slow growers that are only second to *MTBC* bacilli in terms of their ability to infect immune compromised humans and are classically termed as ‘pathogens’. Besides, they are believed to be responsible for Crohn's disease in humans and John's disease in ruminants, especially dairy cattle, causing extensive economic loses to farmers. *MAIC* bacilli, though formally divided into *M. avium* subsp *avium* (*MAA*), *M. avium* subsp *paratuberculosis* (*MAP*) and *M. intracellulare*, remain a challenge to mycobacterial taxonomy due to high heterogeneity within their constituent species. Although the evolution of highly niche adapted parasitic forms by genomic downsizing is an accepted norm in *MTB* [24], [32], the details remain obscure for *MAIC* bacilli. The adaptation of an organism to parasitic lifestyle in a particular host renders various metabolic gene functions redundant due to non-functionalization or pseudogenization of some genes or deletion of the large genomic chunks and the host machinery is utilized to cater these metabolic needs. *M. leprae*, *Shigella flexneri* and *Salmonella typhi* have undergone similar miniaturization by extensive genome reduction [33]. The genome content could, as per this analogy, be inversely proportional to the fitness of the organism in animal hosts. When we look at the genetic similarity of *MAIC* or *MAPC* bacilli to *MIP* in the light of its genome size, it is evident that the former are highly pathogenic and evolved organisms that have shed genes detrimental to pathogenic lifestyle in particular niches.

Since mycobacteria have a restricted synonymous polymorphism rate [15], this implies that deletion or acquisition of the genes might be a more important mechanism than point mutations for generating niche specific evolutionary novelties. The evidence has emerged for lateral gene acquisitions unique to *MAC*, absent in *MTB* and *MAP*, facilitating its intracellularization in protozoic hosts [34]. Similarly, a significant chunk (∼4%) of *MAP* genome is reportedly shaped up by lateral gene acquisitions [35]. Interestingly, *MIP* and *MAA* harbor about 60% and 75% (E<10^−5^) of the genes reported to be laterally acquired in *MAP*, most of them proteobacterial in origin (Saini *et al*, unpublished). These findings are consistent with the ability of *MAIC* bacilli to survive in an extensive range of habitats including soil and water despite their extreme genetic homogeneity and a low rate of recombination. The presence of laterally acquired gene homolog of *rsb*R, a possible sigma B regulator, in *MIP and MAIC* might be seen as one of the plausible mechanisms to overcome stress as this gene has been found to be a positive regulator of *sig*B under stress conditions in *B*. *subtilis* [36]. On the other hand although *MTBC* bacilli appear to be relatively non-amenable to lateral gene acquisition [37], yet only *M. marinum* and *M. ulcerans*, the early pathogenic branch members (“specialists”), were found to possess significant proportions of these genes reported to be laterally acquired in *MAP* (13% and 7.5%, respectively, data not shown). The proteobacterial origins of such major chunk of genome in *MIP* and *MAIC* bacilli and a similar RD profile of these bacilli with respect to early pathogenic lineage of *MTBC* envisage plausible similarities in early life style of the ‘generalist’ and ‘specialist’ groups. Since lateral gene transfers are influenced by physical proximity, it is tempting to speculate that earliest pathogenic and non-pathogenic members of *Mycobacterium* might have shared a common aquatic phase before diversification [38] (Figure 4).

Our findings, thus, in the light of above discussion provide a novel perspective of mycobacterial evolution and we hypothesize that the progenitors of *MAIC* and *MTBC* could have been soil dwelling microbes like *MIP* that preferred water bodies, probably due to nutritional needs and shared a common aquatic phase in their early life history. These bacilli subsequently augmented their fitness for niche specific adaptations by tuning their genomic repertoires with a constant genetic flux aided by extensive and selective lateral gene acquisitions counter balanced by a directional genome decay concomitant with their wide host range. Also, in the backdrop of *MIP*'s congruent RD profile with opportunistic *MAIC,* it appears that immunomodulation in *MIP*, unlike *M. bovis* BCG, may not be due to selective deletion events but rather it is the selective acquisition of genes that shaped up its antigenic repertoire. It, therefore, becomes perceptible that *MIP* existed in nature much before the divergence of *MAIC* and *MAPC* and, therefore, could well be the ancestor of the *MAIC* bacilli that did not parasitize human or animal niches. It has not escaped our notice that the genetic similarity between *MIP* and *MAP* immediately suggests a plausible advantage for therapeutic intervention against Crohn's and Johne's diseases.

## Materials and Methods

### Bacterial cultures and DNA samples

*MIP* stock culture was a kind gift from Rajani Rani, National Institute of Immunology, New Delhi, India. The bacteria were streaked onto Middlebrook (MB) 7H11 agar plate supplemented with 1xOADC. Culture was also streaked onto LB agar to check for any contaminating bacteria. Once the purity of the culture was confirmed [14], *MIP* genomic DNA was isolated from culture grown in Middlebrook (MB) 7H9 liquid medium supplemented with 0.5% glycerol, 0.2% Tween-80, 1xADC with constant shaking (200 rpm) at 37°C. The genomic DNA of *MTB* was obtained from the RIVM, Bilthoven, Netherlands and the DNA of several other non-tuberculous mycobacteria (NTMs) such as *M. avium, M. intracellulare, M. smegmatis, M. xenopi, M. abscessus, M. fortuitum/peregrinum, M. scrofulaceum, M. fortuitum, M. gordonae, M. paratuberculosis, M. marinum and M. kansasii* were a gift from Leonardo A. Sechi, Dept. of Biomedical Sciences, University of Sassari, Italy.

### DNA fingerprinting and genotyping

DNA fingerprinting by fluorescent amplified fragment analysis was performed as described previously [16], [39]. Briefly, the profiling of whole genome micro-restriction fingerprints with *Eco*RI/*Mse*I enzymes using fluorescence tagged primer pairs *Eco*RI+A/*Mse*I+0 and *Eco*RI+G or A / *Mse*I+0 was performed for all the strains. The PCR amplified fragments for each of the strains were then subjected to electrophoretic separation on a 5% acrylamide gel and scoring of the fluorescent markers was done using an automated DNA analysis workstation (ABI Prism 3100 DNA sequencer).

ERIC based genotyping was carried out by PCR using primers ERIC1R (5-ATGTAAGCTCCTGGGGATTCAC) and ERIC2 (5-AAGTAAGTGACTGGGGTGAGCG). Amplification reactions were performed in a total volume of 20 µl containing 50 ng of DNA, 1X PCR buffer (Applied Biosystems), 2 mM MgCl_2_ (Applied Biosystems), 200 mM deoxynucleoside triphosphate, 20 picomoles of each primer and 2 U of Taq DNA polymerase (Applied Biosystems, USA). The reaction mixtures were incubated in GeneAmp PCR system 9700 for 2 min at 94°C, followed by 35 cycles of 94°C for 45 s, 52°C for 1 min, and 70°C for 10 min and a final extension at 70°C for 20 min as described previously [17].

### Comprehensive phylogeny and comparative genomics

A comprehensive genomic characterization of *MIP* was carried out based on several molecular signatures: i) FAFLP based high resolution fingerprinting [16], ii) ERIC typing [17], iii) sequencing of candidate orthologues corresponding to *rrn*16, *hsp*65, *sod*A, *rpo*B, *gyr*B, *rec*A, *ITS* and the 32 kDa protein coding gene [18], [19], [20] iv) presence/absence of RDs [24], [32], and, v) *kat*G and *gyr*A polymorphisms [15]. With respect to these features, *MIP* was compared against 9 genome sequences of mycobacteria including *M. marinum, M. ulcerans, M. tuberculosis* H37Rv, *M. tuberculosis* CDC1551, *M. bovis*, *M. bovis* BCG, *M. avium* subsp *paratuberculosis*, *M. smegmatis* and *M. avium* subsp *avium*. The genomes were downloaded from NCBI (ftp://ftp.ncbi.nih.gov/genomes/Bacteria/). Phylogenetic trees based on the candidate gene polymorphisms in either individual genes or their concatenated ‘super loci’ were obtained by using the neighbour joining method with Kimura-2 parameter (K2P) and a distance correction model with 1000 bootstrap replicates [in MEGA3.1; 40]. In case of FAFLP data, the trees were constructed using the binary data by minimum evolution method with topology validated through an interior branch test, a t-test, which is computed using the bootstrap procedure. ERIC based phylogenetic trees were also obtained based on binary data, but by using the neighbor joining method with nucleotide p distance model with 100 bootstrap replicates [in MEGA3.1; 40]. Internal branch topology was validated *via* the interior branch test.

### Whole-genome sequencing

The shotgun data for *MIP* genome were generated from paired end sequences from whole genome shot gun libraries with an average insert size of 2–3 kb and 4–5 kb using BDT (big dye terminator) technology on ABI3700 DNA sequencers. The data were subsequently assembled and analyzed using Phred/Phrap/Consed package available from University of Washington, U.S.A.

## Supporting Information

Figure S1 Phylogenetic trees based on comparison of the DNA sequences corresponding to gyrB gene (A) and 32kDa protein gene (B) of MIP and other mycobacteria. Sequence alignment was performed in Clustal W software and phylogenetic trees were developed in MEGA3.1 using bootstrapping method.
(0.11 MB PPT)

Figure S2 Alignment of gyrB of MIP with other members of MAIC complex (M. avium and M. intracellulare)
(0.15 MB PPT)

Figure S3 Alignment of 32kDa protein gene of MIP with other members of MAIC complex (M. avium and M. intracellulare). The identities are depicted by dots only.
(0.12 MB PPT)
