# Supplementary figures and images for: Molecular Analysis of a Leprosy Immunotherapeutic Bacillus Provides Insights into Mycobacterium Evolution
Source: PLoS One. 2007 Oct 3;2(10):e968. doi: 10.1371/journal.pone.0000968 (PMC1989137; doi:10.1371/journal.pone.0000968)

## Slide 1
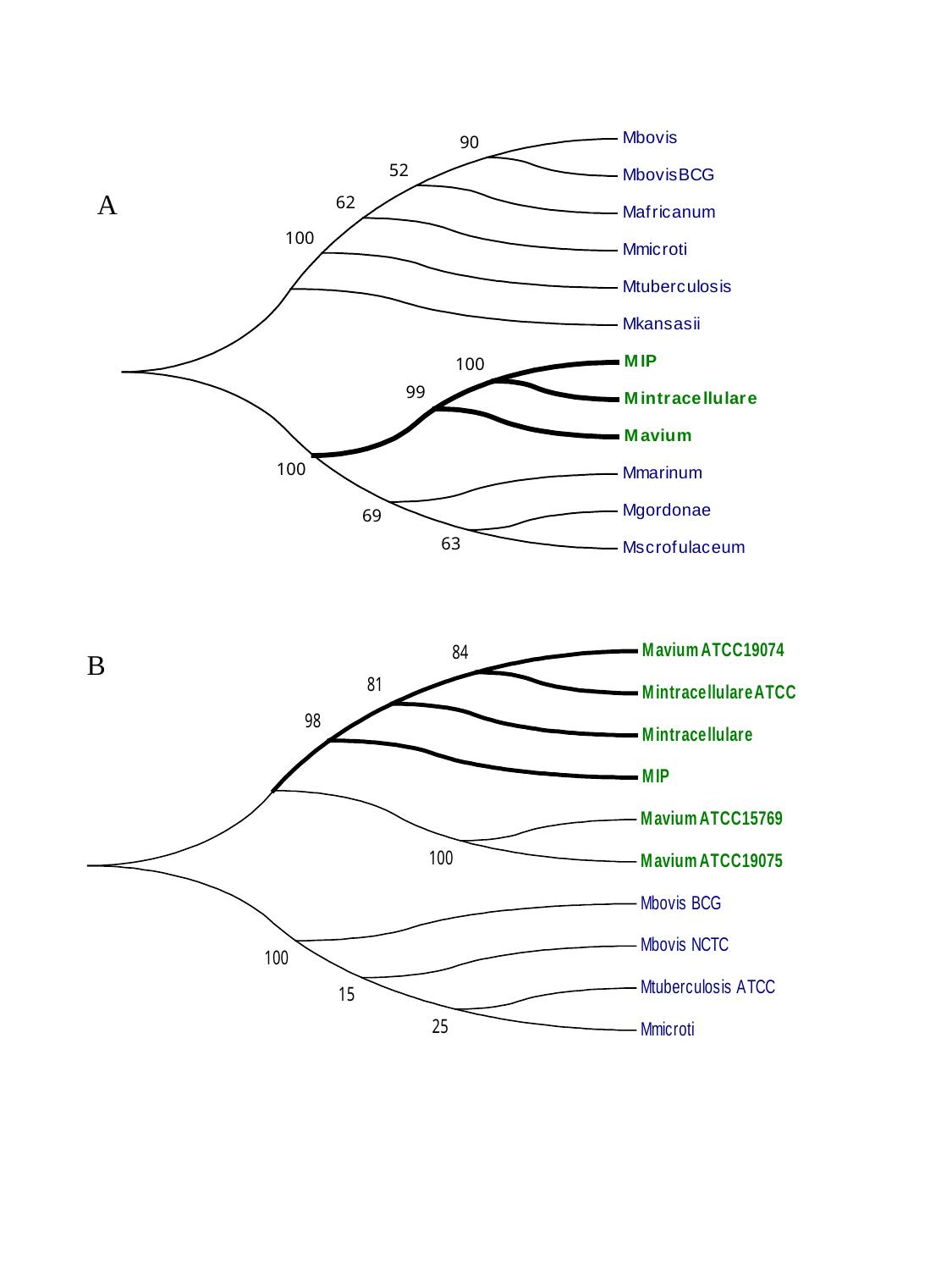

A
B

Supplement: Figure S1 — Phylogenetic trees based on comparison of the DNA sequences corresponding to gyrB gene (A) and 32kDa protein gene (B) of MIP and other mycobacteria. Sequence alignment was performed in Clustal W software and phylogenetic trees were developed in MEGA3.1 using bootstrapping method. (0.11 MB PPT) [file pone.0000968.s001.ppt]

## Slide 1
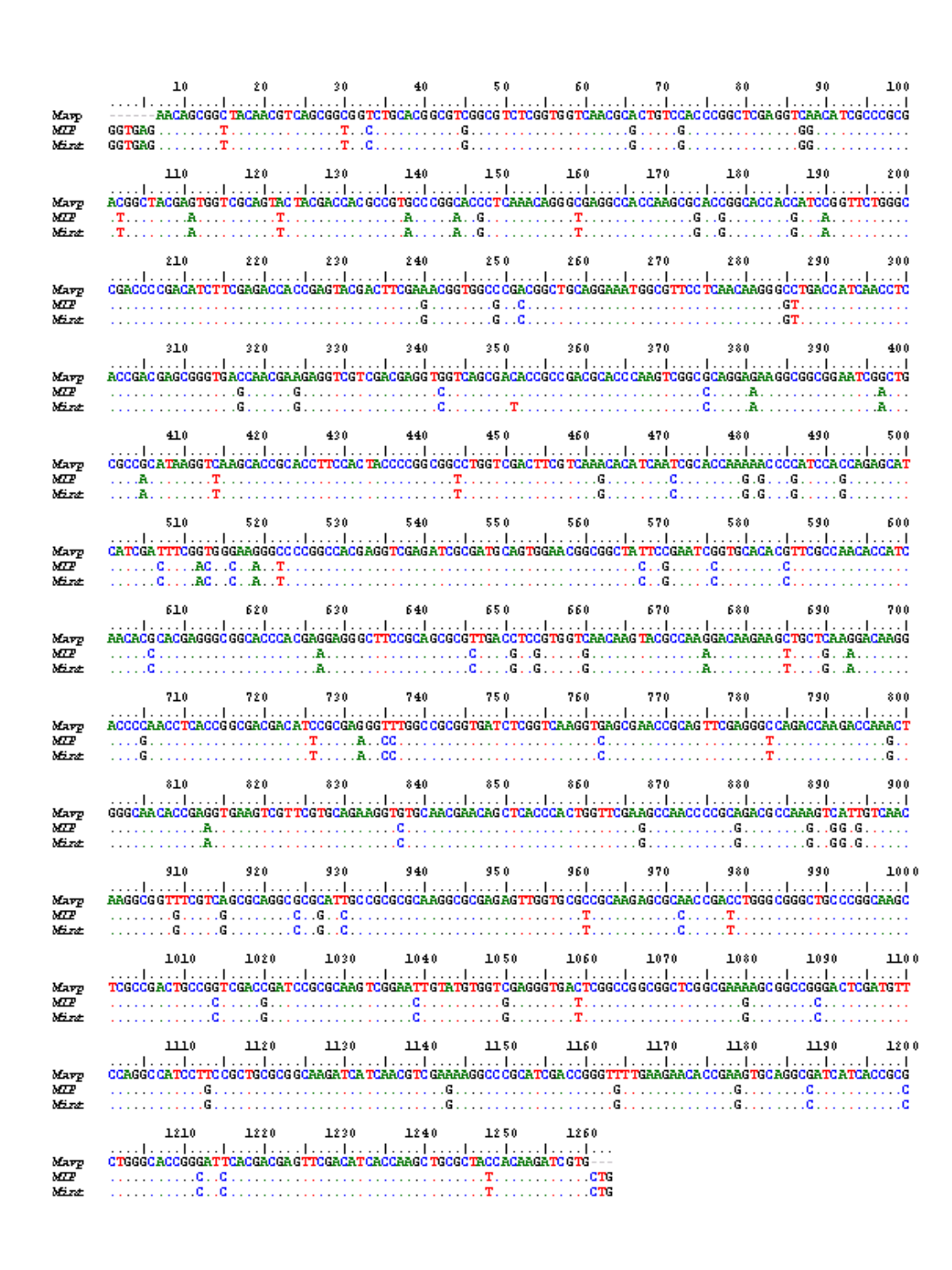

Supplement: Figure S2 — Alignment of gyrB of MIP with other members of MAIC complex (M. avium and M. intracellulare) (0.15 MB PPT) [file pone.0000968.s002.ppt]

## Slide 1
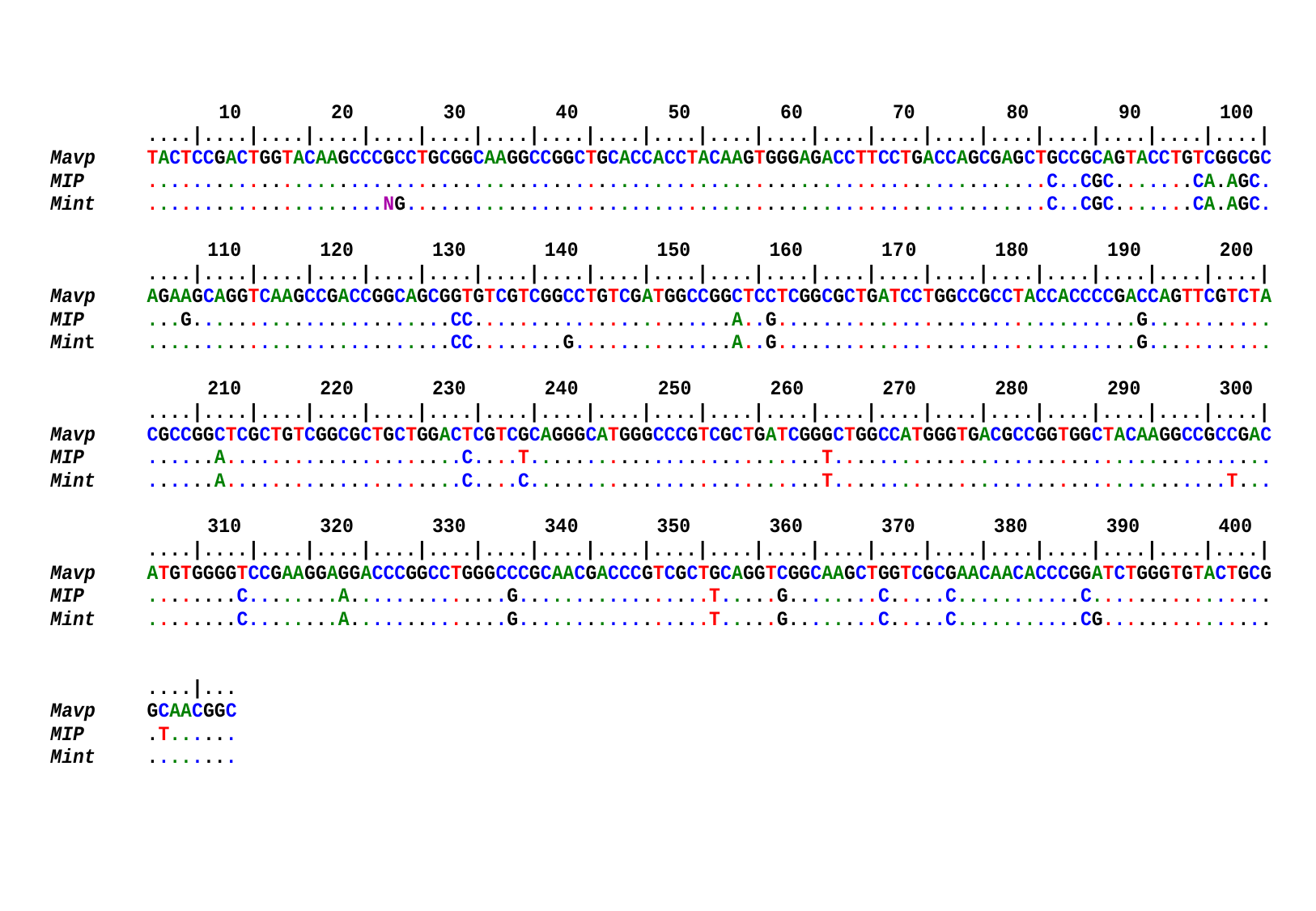

Supplement: Figure S3 — Alignment of 32kDa protein gene of MIP with other members of MAIC complex (M. avium and M. intracellulare). The identities are depicted by dots only. (0.12 MB PPT) [file pone.0000968.s003.ppt]
